# Functional analysis of fatty acid binding protein 7 and its effect on fatty acid of renal cell carcinoma cell lines

**DOI:** 10.1186/s12885-017-3184-x

**Published:** 2017-03-14

**Authors:** Naohisa Takaoka, Tatsuya Takayama, Seiichiro Ozono

**Affiliations:** 1 Department of Urology, Hamamatsu University School of Medicine, 1-20-1 Handayama, Higashi-ku, Hamamatsu, Shizuoka, 431-3192 Japan; 2 Department of Urology, Jichi Medical University, 3311-1 Yakushiji, Shimotsuke, Tochigi, 329-0498 Japan

**Keywords:** FABP7, Renal cell carcinoma, Subculture, Cell proliferation, Cell migration, Docosatetraenoic acid

## Abstract

**Background:**

Renal cell carcinomas (RCCs) overexpress fatty acid binding protein 7 (FABP7). We chose to study the TUHR14TKB cell line, because it expresses higher levels of FABP7 than other cell lines derived from renal carcinomas (OS-RC-2, 786-O, 769-P, Caki-1, and ACHN).

**Methods:**

FABP7 expression was detected using western blotting and real-time PCR. Cell proliferation was determined using an MTS assay and by directly by counting cells. The cell cycle was assayed using flow cytometry. Cell migration was assayed using wound-healing assays. An FABP7 expression vector was used to transfect RCC cell lines.

**Results:**

The levels of FABP7 expressed by TUHR14TKB cells and their doubling times decreased during passage. High-passage TUHR14TKB cells comprised fewer G0/G1-phase and more S-phase cells than low-passage cells. Cell proliferation differed among subclones isolated from cultures of low-passage TUHR14TKB cells. The proliferation of TUHR14TKB cells decreased when FABP7 was overexpressed, and the cell migration property of TUHR14TKB cells were decreased when FABP7 was overexpressed. High concentrations of docosatetraenoic acid and eicosapentaenoic acid accumulated in TUHR14TKB cells that overexpressed FABP7, and docosatetraenoic acid enhanced cell proliferation.

**Conclusions:**

The TUHR14TKB cell line represents a heterogeneous population that does not express FABP7 when it rapidly proliferates. The differences in FABP7 function between RCC cell lines suggests that FABP7 affects cell proliferation depending on cell phenotype.

**Electronic supplementary material:**

The online version of this article (doi:10.1186/s12885-017-3184-x) contains supplementary material, which is available to authorized users.

## Background

Kidney cancer is the 15th most common malignancy worldwide. In 2008, approximately 271,000 new cases were diagnosed, and 116,000 patients died from this disease [1]. These rates are approximately twice as high in men as in women [1]. Renal cell carcinomas (RCCs) represent 91.6% of kidney cancers [2]. The identification of molecular markers in body fluids, which can be used for screening, diagnosis, follow-up, and monitoring drug-based therapy of patients with RCC, is one of the most important challenges of cancer research [3]. In a search for candidate markers of RCC, we identified the gene (*FABP7*) encoding fatty acid binding protein 7 [4].

Human *FABP7* was first isolated from a library of fetal brain complementary DNA (cDNA), and the *FABP7* transcript is expressed specifically in adult human brain and skeletal muscle [5]. Further, *FABP7* is expressed more abundantly during the early stages of maturation of the brain [5]. RCCs overexpress FABP7 [4, 6–14], and *FABP7* transcripts are present in the tumors or urine of patients with RCC [9]. The role of FABP7 in inhibiting the proliferation of a breast cancer cell line suggests that it may act as a tumor suppressor [15, 16]. In apparent contradiction to this, inhibition of FABP7 expression by small interfering RNAs (siRNAs) significantly reduces the proliferation of certain human cancer cell lines [17–21], and overexpression of FABP7 stimulates the proliferation of RCC cell lines [14]. Further, inhibition of FABP7 expression by siRNAs significantly decreases the ability of certain human cancer cell lines to migrate [17–19, 21–23]. Moreover, FABP7 enhances the migration of glioma cells [24], and an antibody against FABP7 inhibits cell migration [25].

To better understand the role of FABP7 in RCC and to attempt to resolve the conflicting findings summarized above, the present study aimed to analyze the effects of FABP7 on the phenotypes of RCC cell lines, with particular focus on the composition of the fatty acids accumulating in cell lines that overexpress FABP7.

## Methods

### Reagents

Reagents and their sources were as follows: RPMI 1640 medium, Oligo(dT)_12–18_ Primer, SuperScript® III Reverse Transcriptase, SYBR® Green PCR Master Mix, pENTR™/D-TOPO® vector, Gateway® pT-Rex™-DEST30 vector, pT-Rex/GW-30/lacZ vector, pcDNA™6/TR vector, Lipofectamine® 2000 Transfection Reagent and blasticidin S HCl (Thermo Fisher Scientific, Waltham, MA, USA); docosatetraenoic acid, eicosapentaenoic acid (EPA) (NU-CHEK PREP, Inc.; Elysian, MN, USA); oligopeptides (Hokkaido System Science, Sapporo, Hokkaido, Japan); Tris, dithiothreitol, sodium orthovanadate, phenylmethanesulfonyl fluoride, and doxycycline hyclate (Sigma-Aldrich, St. Louis, MO, USA); sodium chloride (Nacalai Tesque, Kyoto, Japan); EDTA, sodium deoxycholate, sodium fluoride, sodium dodecyl sulfate (SDS), 4% paraformaldehyde and crystal violet (Wako, Osaka, Japan); IGEPAL CA-630 (MP Biomedicals, Santa Ana, CA, USA); protease inhibitor cocktail tablet (Complete, Mini, EDTA-free), geneticin (G418) (Roche Diagnostics GmbH, Mannheim, Germany); and SacI, XhoI (Takara Bio Inc., Otsu, Shiga, Japan).

### Cell culture

The 786-O cell line (CRL-1932) was purchased from the American Type Culture Collection (Manassas, VA, USA). The TUHR14TKB cell line (RCB1383) was provided by RIKEN (Tsukuba, Ibaraki, Japan). Short tandem-repeat typing was performed to confirm the identity of high-passage TUHR14TKB cells, and the data were verified using the RIKEN short tandem-repeat database [26]. All cell lines were grown in RPMI 1640 medium supplemented with 10% (*v*/v) or 1% fetal bovine serum (FBS) (Nichirei Biosciences Inc., Tokyo, Japan). Cells were cultured at 37 °C in a humidified atmosphere containing 5% CO_2_. Docosatetraenoic acid or EPA (100 mM each) was dissolved in ethanol, and a 1:2000 dilution of each fatty acid was added to the culture medium.

### Cell cloning

Clones were isolated from low-passage cultures of TUHR14TKB cells by plating the cells at limiting dilution in 96-well plates. The cells were serially diluted to 128 to 4 viable cells/mL, and 50 μL was added per each well of a 96-well plate. After incubation at 37 °C in a humidified atmosphere containing 5% CO_2_, single colonies in the wells were expanded.

### Real-time PCR analysis

Real-time PCR assays were performed using a modified version of the method described by Takaoka et al. [27]. Cells were cultured in 10-cm dishes. Total RNA was isolated from cultured cell lines using the RNeasy Mini Kit (QIAGEN, Hilden, Germany) according to the manufacturer’s instructions. Two micrograms of RNA was reverse transcribed using SuperScript® III Reverse Transcriptase primed by 500 ng of Oligo(dT)_12–18_ Primer according to the manufacturer’s protocol. Real-time PCR analysis of *FABP7* expression was performed using an Applied Biosystems StepOnePlus (Thermo Fisher Scientific). The final PCR reaction mix (20 μL) included 2 μL of each specific primer (5 μM), 1 μL of first-strand cDNA, and 10 μL of SYBR® Green PCR Master Mix. Plasmids that encode FABP7 and TATA box binding protein (TBP) were synthesized as described previously [27], and standard curves for each gene were generated using seven serial dilutions of plasmid templates (0.1 nM to 0.1 fM). TBP was used as an internal control. Takaoka et al. [27] and Jung et al. [28] reported the sequences of the primers used to amplify FABP7 and TBP, respectively.

### Western blotting

Western blotting was performed using a modified version of a published method [27]. Cells were cultured in 6-well culture plates or in 10-cm culture dishes. The cells were detached using trypsin-EDTA, collected by centrifugation, and washed once with phosphate-buffered saline (PBS). The pellets were lysed on ice for 30 min in RIPA buffer (50 mM Tris, pH 8.0, 150 mM sodium chloride, 5 mM EDTA, 0.5% sodium deoxycholate, 1% IGEPAL CA-630, and 0.1% SDS) containing 2 mg/L sodium orthovanadate, 10 mM sodium fluoride, 1 mM phenylmethanesulfonyl fluoride, 2 mM dithiothreitol, and a protease inhibitor cocktail tablet. Lysates were centrifuged for 10 min at 4 °C at 18,000×g. The supernatants were transferred to sterile microcentrifuge tubes. Protein concentrations were determined using the Bio-Rad Protein Assay Kit II (Bio-Rad, Hercules, CA, USA). Cell extracts (20 μg) were electrophoresed through an 18% (*w*/*v*) polyacrylamide-SDS gel. The proteins were transferred electrophoretically onto a PVDF membrane (GE Healthcare UK Ltd., Little Chalfont, Buckinghamshire HP7 9NA, England), and the membrane was incubated with 1 g/L of an FABP7 antibody (AF3166; R&D Systems, Minneapolis, MN, USA) diluted 1:5000. Antibody-antigen complexes were visualized using peroxidase-conjugated anti-goat IgG (86,285; Jackson ImmunoResearch Laboratories, West Grove, PA) and Immobilon Western HRP Substrate (Millipore, Billerica, MA, USA). A mouse monoclonal anti-α-tubulin antibody (T6074; Sigma-Aldrich, St. Louis, MO, USA) served as an internal control.

### Flow cytometry

Cells were plated in 10-cm culture dishes at a density of 2 × 10^6^ cells per plate and incubated for two days at 37 °C in an atmosphere containing 5% CO_2_. After incubation, the cells were harvested with trypsin/EDTA, washed once with PBS, and then resuspended to 1 × 10^6^ cells/0.2 mL in PBS containing 0.25% Triton X-100 for 5 min at room temperature. Cellular DNA in each cell suspension was stained using 0.6 mL of 50 mg/L propidium iodide for 10 min at room temperature. Cell-cycle analysis was performed using an EPICS-XL flow cytometer (Beckman-Coulter, Brea, CA, USA).

### Vector construction

To generate FABP7 expression constructs, the FABP7 cDNA sequence was amplified using PCR with the primers Full B-FABP F2 (5′-CACCATGGTGGAGGCTTTCTGT) and Full B-FABP R3 (5′-TTATGCCTTCTCATAGTGGCG). The PCR product was inserted into pENTR™/D-TOPO® via TOPO cloning (Invitrogen, CA, USA). The cloning vector (pENTR-FABP7) was transferred to the Gateway® pT-Rex™-DEST30 vector via gateway recombination (Invitrogen, CA, USA). The plasmid generated (DEST30-FABP7) was verified by direct DNA sequencing. The pT-Rex/GW-30/lacZ vector that expresses β-galactosidase (lacZ) served as a control.

### Transfection

TUHR14TKB and 786-O cells were transfected with 2 μg of XhoI-digested pcDNA6/TR using FuGene® HD transfection reagent (Promega, Madison, WI, USA). The transfectants were cultured in RPMI 1640 medium containing 10% FBS and 5 mg/L blasticidin S HCl. The pcDNA6™/TR transfectants (TUHR-TR, TUHR14TKB pcDNA6™/TR transfectant; 786-O TR, 786-O pcDNA6™/TR transfectant) were expanded and transfected in the presence of Lipofectamine® 2000 transfection reagent with 4 μg of DEST30-FABP7 or the empty vector pT-Rex/GW-30/lacZ digested with SacI. The transfectants were cultured in RPMI 1640 medium containing 10% FBS, 5 mg/L blasticidin S HCl, and 0.3 g/L G418, and blasticidin- and G418-resistant cells were expanded. The FABP7 or control-vector transfectants of TUHR-TR or 786-O TR were cultured in RPMI 1640 medium containing 10% FBS or 1% FBS with 5 mg/L blasticidin S HCl, 0.3 g/L G418, and 1 mg/L doxycycline hyclate for one to three days and then subjected to western blotting or the following assays: MTS, cell counting, or wound-healing. SRL Inc. (Tokyo, Japan) performed the analyses of the fatty acid composition of the TUHR-TR transfectants.

### Cell proliferation assay

Cells were plated in 96-well cell culture plates at 400 (786-O transfectant) or 2000 cells per well (low-passage or high-passage TUHR14TKB or TUHR14TKB transfectants, respectively) in 100 μL of culture medium. The plates were incubated at 37 °C in an atmosphere containing 5% CO_2_. The cells were analyzed using a CellTiter 96® AQueous One Solution Cell Proliferation Assay Kit (Promega, Madison, WI, USA) according to the manufacturer’s instructions. Absorbance (490 nm) was measured one, two, and three days after cell plating. Doubling times were determined from four replicate samples per point.

### Cell counts

TUHR14TKB transfectants were plated in 24-well cell culture plates (10,000 cells per well) in 500 μL of RPMI 1640 medium containing 10% FBS with 5 mg/L blasticidin S HCl, 0.3 g/L G418, and 1 mg/L doxycycline hyclate. The plates were incubated at 37 °C in an atmosphere containing 5% CO_2_. Cells on the plate were fixed with 4% paraformaldehyde and stained with 0.1% crystal violet one, two, and three days after plating. The numbers of cells were counted in five random fields using a light microscope (×100).

### Wound-healing assay

Cells (1 × 10^6^) were seeded in 24-well plates. After incubation overnight (786-O TR transfectant) or for one day (TUHR-TR transfectant), an artificial wound was created (0 h) using a 200-μL tip to introduce a gap in the confluent cell monolayer, and the culture medium was changed. Images were acquired at 0 h and 6 h (786-O TR transfectant) or 16 h (TUHR-TR transfectant). The wounded areas were measured before and after healing.

### Data analysis

Cell proliferation and migration data were analyzed using the Student *t* test. Statistical significance was defined as *p* < 0.05.

## Results

### Analyses of FABP7 expression and proliferation of TUHR14TKB cells during passage in culture

**Fig. 1:**
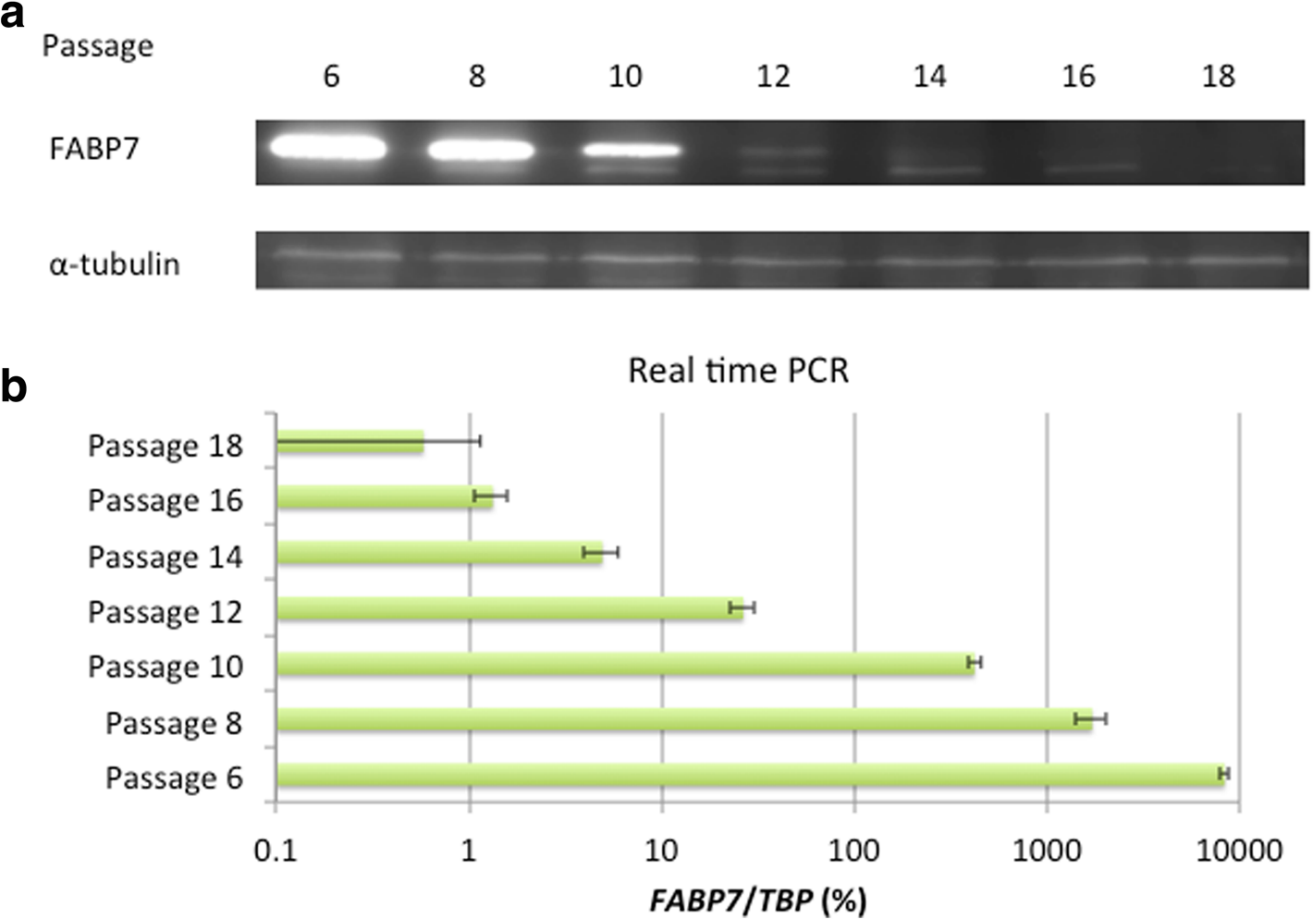
Expression of FABP7 during subculture of TUHR14TKB cells. The zero passage (0) was started when the cells were received from RIKEN. TUHR14TKB cells were cultured in RPMI 1640 medium containing 10% FBS for one to two weeks, harvested when they reached confluence, and assayed for FABP7 expression. **a** Western blot analysis of FABP7 expression. **b** Real-time PCR analysis of FABP7 expression

**Fig. 2:**
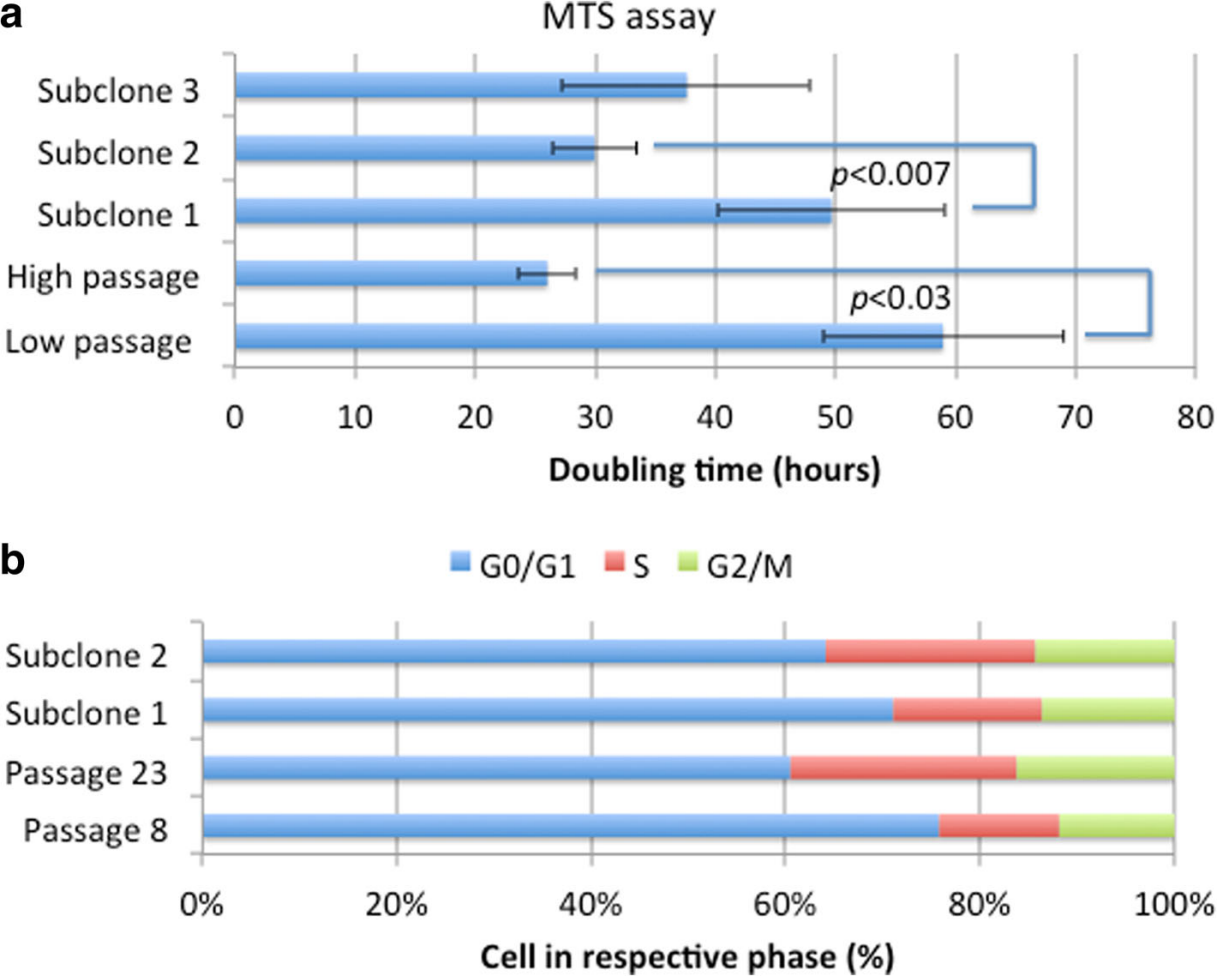
Proliferation of TUHR14TKB cells during passage. **a** The doubling times of TUHR14TKB cells and its subclones were subjected to MTS assay. Low and high passages are defined as TUHR14TKB cells 7–9 and 19–23 passages, respectively. “Subclones 1, 2, and 3” represents subclones from low-passage TUHR14TKB cells. The assay was repeated three to five times, and the data represent the average value and standard deviation (error bars). **b** The stages of the cell cycle of low- and high-passage TUHR14TKB cells and the subclones were determined using flow cytometry

High levels of FABP7 were detected during passages 6–8 of TUHR14TKB cells, but not during passages 16–18 (Fig. 1a). The levels of *FABP7* expressed by TUHR14TKB cells decreased by approximately four-fold between two cell passages (Fig. 1b). In contrast, the doubling time of low-passage cells was approximately twice that of high-passage cells (Fig. 2a). The doubling times differed among cells that were isolated from individual colonies of low-passage TUHR14TKB cells (Fig. 2a). Further, the percentage of S-phase cells in high-passage TUHR14TKB cells increased and was accompanied by a decrease in the percentage of G0/G1-phase cells compared with low-passage TUHR14TKB cells (Fig. 2b).

### Functional analysis of FABP7 in RCC cells

**Fig. 3:**
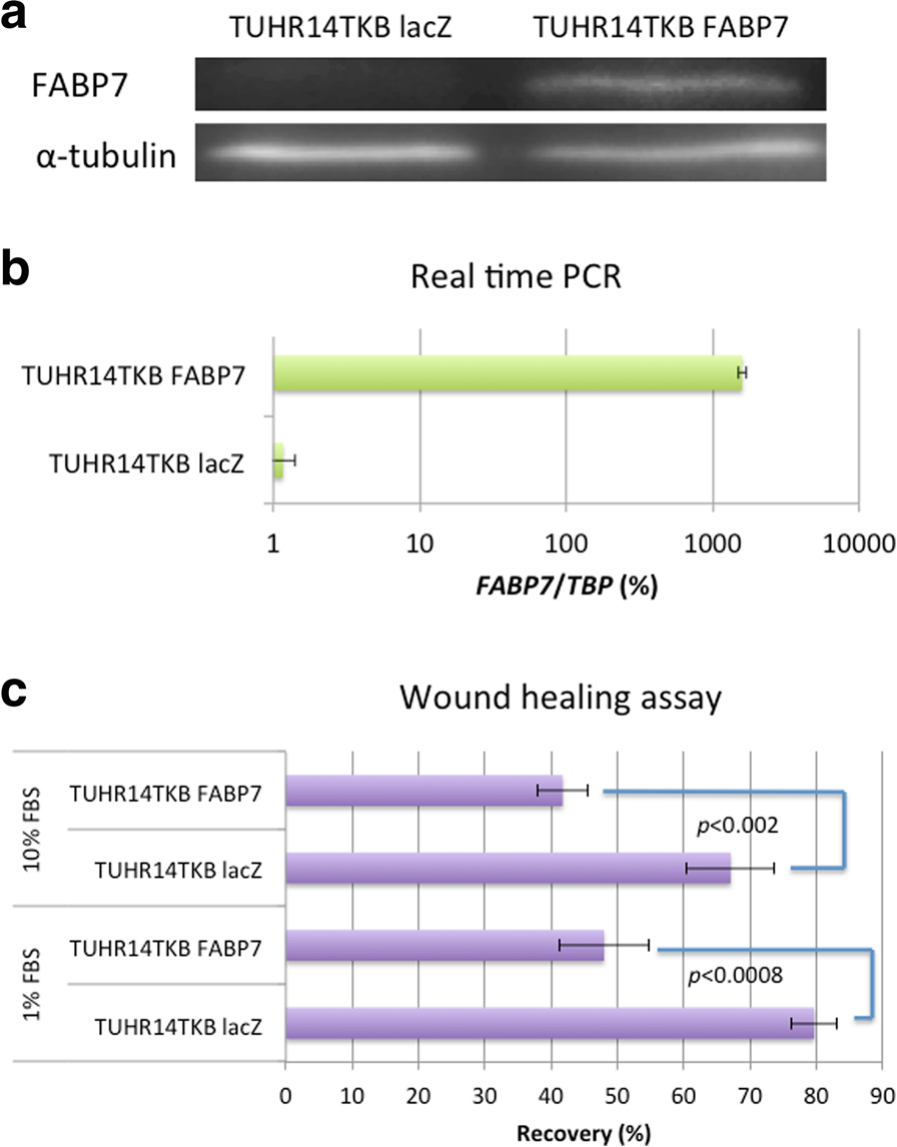
Effect of FABP7 on the migration of TUHR14TKB cells. TUHR14TKB cells were transfected with the FABP7 or lacZ expression vector. **a** Western blot analysis of FABP7 expression by cells transfected with the FABP7 vector or control (lacZ) vector. **b** Real-time PCR analysis of FABP7 expression in cells transfected with the FABP7 vector or lacZ vector. **c** Wound-healing assays. TUHR14TKB transfectants were cultured in RPMI 1640 medium containing with 10% FBS or 1% FBS with 5 mg/L blasticidin S HCl, 0.3 g/L G418, and 1 mg/L doxycycline hyclate. The data represent the average and standard deviation (error bars) of four experiments

**Fig. 4:**
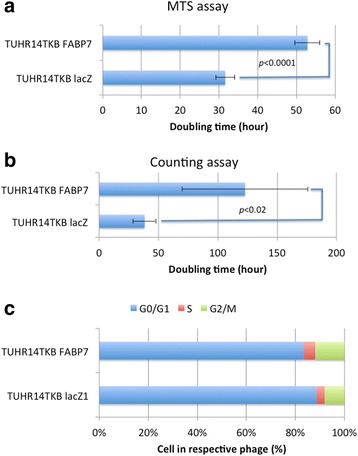
Effect of FABP7 on cell proliferation and cell cycle of TUHR14TKB cells. TUHR14TKB cells were cultured in RPMI 1640 medium containing 10% FBS, 5 mg/L blasticidin S HCl, 0.3 g/L G418, and 1 mg/L doxycycline hyclate. Assays to determine the rate of cell proliferation: **a** MTS assay. The data represent the average and standard deviation (error bars) of five experiments. **b** Cell counts. The data represent the average and standard deviation (error bars) of four experiments. **c** The stages of the cell cycles of TUHR14TKB FABP7 and TUBR14TKB lacZ were determined using flow cytometry

We transfected FABP7 low-expressing TUHR14TKB and 786-O cells with an FABP7 expression vector (Fig. 3a and b and Additional file 1: Figure S1a and S1b). In the presence of 10% FBS, the doubling time of TUHR14TKB cells that overexpressed FABP7 was significantly longer than that of cells transfected with the control vector (Fig. 4a and b). Although TUHR14TKB cells transfected with the control vector were able to proliferate, the cells that overexpressed FABP7 were unable to proliferate in the presence of 1% FBS (Additional file 2: Figure S2). Further, the percentage of TUHR14TKB FABP7 in G2/M increased compared with that of TUHR14TKB lacZ cells (Fig. 4c), indicating that FABP7 induced the arrest of TUHR14TKB in G2. In contrast, overexpression of FABP7 stimulated the proliferation of the 786-O cell line cultured in medium containing 1% FBS (Additional file 1: Figure S1c).

Wound-healing assays revealed that TUHR14TKB cells that overexpressed FABP7 migrated significantly slower than TUHR14TKB cells transfected with the control vector (Fig. 3c), although overexpression of FABP7 did not affect the migration of 786-O cells (Additional file 1: Figure S1d).

### Effects of fatty acids on TUHR14TKB cells expressing FABP7

**Fig. 5:**
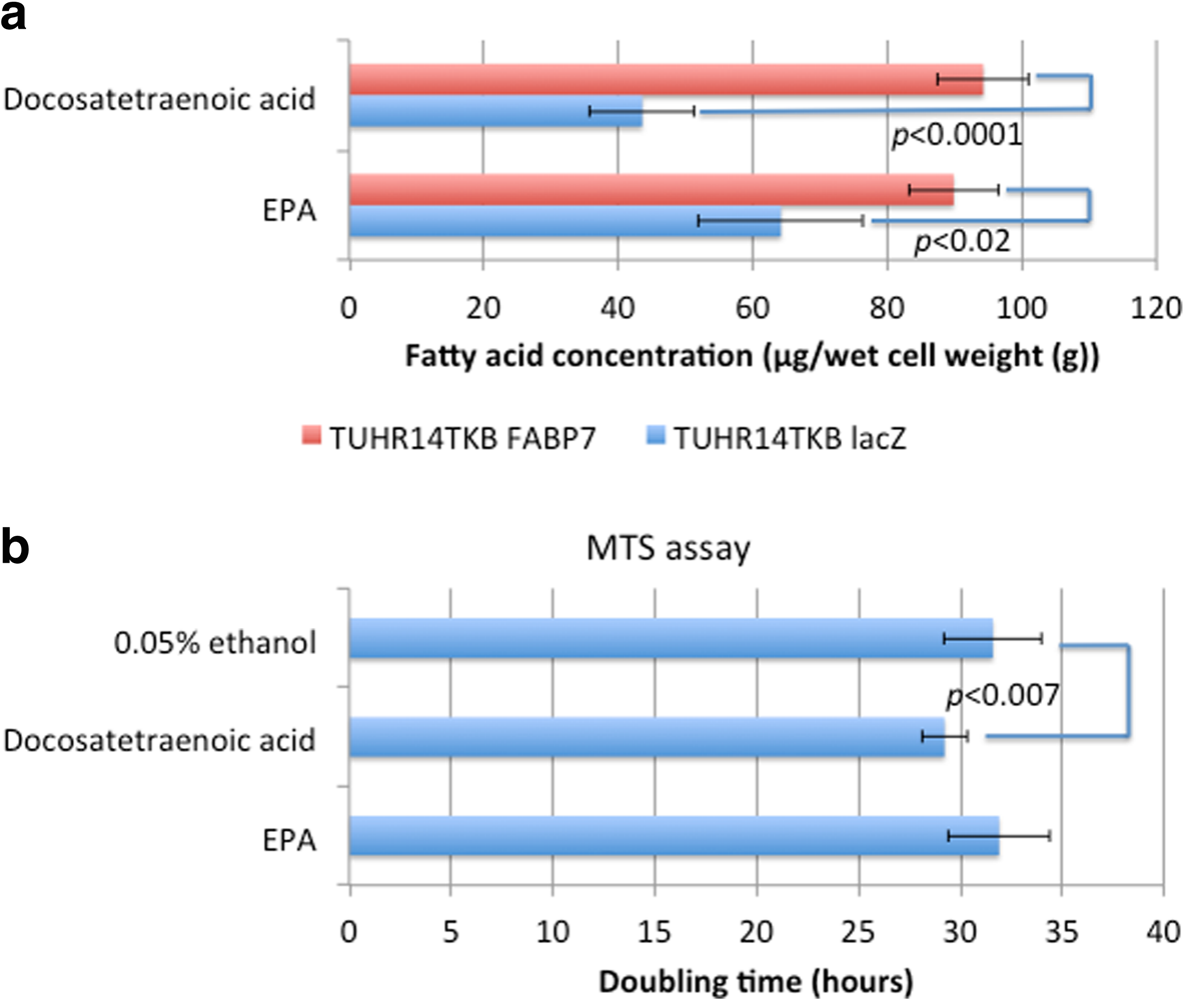
Effect of fatty acids on cell proliferation. **a** Docosatetraenoic acid or EPA concentration of TUHR14TKB cells transfected with the FABP7 or lacZ expression vector. Four independent cell cultures were harvested, and the concentration of docosatetraenoic acid or EPA was determined. The data represent the average and standard deviation. **b** Cell proliferation was assayed in the presence or absence of 50 μM docosatetraenoic acid or EPA in RPMI 1640 medium containing 10% FBS, 5 mg/L blasticidin S HCl, 0.3 g/L G418, and 1 mg/L doxycycline hyclate. The doubling times of TUHR14TKB lacZ cells were determined using an MTS assay. The data represent the average and standard deviation (error bars) of 12 experiments

Although FABP7 binds to fatty acids, it does not catalyze de novo fatty acid synthesis, suggesting that FABP7 expression leads to the accumulation of fatty acid in cells. Docosatetraenoic acid and EPA accumulated in TUHR14TKB cells that expressed FABP7 (Fig. 5a). In contrast, other fatty acids did not accumulate in TUHR14TKB cells that expressed FABP7 (Additional file 3: Table S1). Therefore, we tested the effects of docosatetraenoic acid or EPA on the proliferation of TUHR14TKB cells. The addition of docosatetraenoic acid significantly stimulated the proliferation of TUHR14TKB cells that expressed β-galactosidase (Fig. 5b).

## Discussion

Human RCCs overexpress FABP7 [4, 6–14], indicating that FABP7 might affect the progression of RCC. Therefore, we studied FABP7 function using RCC cell lines. In the present study, we show that the levels of FABP7 dramatically decreased during passage of the RCC cell line TUHR14TKB. Further, FABP overexpression differentially affected the proliferation of the RCC cell lines analyzed here. Thus, overexpression of FABP7 decreased the proliferation of TUHR14TKB cells. In contrast, overexpression of FABP7 increased the proliferation of 786-O cells.

*FABP7* transcripts are expressed in 18 of 30 clear cell-type RCC lesions but in only 4 of 19 RCC cell lines [6]. These results are consistent with our previous findings that FABP7 is expressed in one (TUHR14TKB) of six RCC cell lines [27]. We show here that the levels of FABP7 decreased during the passage of TUHR14TKB cells (Fig. 1). Further, TUHR14TKB cells proliferated faster during continued passage (Fig. 2a), suggesting that continued passage selected for cells that did not express FABP7 and therefore proliferated at an increased rate. Moreover, the doubling times of subclones of TUHR15TKB cells differed significantly (Fig 2a), which is consistent with the loss of FABP7 expression during attempts to establish cell lines from primary RCC tumor tissue. In addition, glioblastoma neurospheres express FABP7 at higher levels than those of adherent cells derived from the same tumor [21]. Therefore, conditions that favor the formation of spheres may provide a selective advantage for primary RCC cells that express FABP7.

Overexpression of FABP7 inhibited the proliferation of TUHR14TKB cells (Fig. 4a and b), which is consistent with findings that FABP7 (referred to formerly in the studies cited here as the protein encoded by mammary-derived growth inhibitor-related gene) inhibits the proliferation of breast cancer cell lines [15, 16]. Further, high tumor-grade (G3 + G4) RCCs express significantly lower levels of *FABP7* mRNA than low-grade (G1 + G2) RCCs [10], and *FABP7* is highly expressed in primary melanomas compared with metastatic melanomas [29, 30]. In contrast, knockdown of FABP7 expression inhibits the proliferation of melanoma cells [17, 18], an RCC cell line [19], a breast cancer cell line [20], and glioblastoma cells [21]. Further, we show here that FABP7 overexpression did not affect proliferation of the 786-O cell line (Additional file 1: Figure S1c and [14]), and down-regulation of FABP7 expression by *FABP7*-specific siRNAs does not affect the proliferation of certain melanoma cells [17]. Interestingly, FABP7 overexpression stimulated the proliferation of 786-O cells in medium containing 1% FBS (Additional file 1: Figure S1c and [14]).

The present and previous studies demonstrate that the effect of FABP7 on cell proliferation varies among cell lines and with cell culture conditions. These findings may be explained by the interaction of FABP7 with molecule(s) that inhibit or enhance cell proliferation. Cancer is a multistage disease, which develops through a succession of mutations [31, 32]. Thus, FABP7 and other molecule(s) may control cell proliferation through a similar mechanism. Another explanation for the inconsistencies among studies of FABP7 function may be that FABP7 modulates signaling networks that influence cell proliferation.

Down-regulation of FABP7 expression by siRNAs significantly reduces the migration of melanoma cell lines [17, 18], an RCC cell line [19], breast cancer cells [20], and malignant glioma cells [21–23]. Further, overexpression of FABP7 enhances the migration of glioma cells [24]. In contrast, FABP7 overexpression inhibited the migration of TUHR14TKB cells that was revealed using a wound-healing assay (Fig. 3c). Thus, the effect of wound healing may be related to the effect of proliferation.

Docosatetraenoic acid and EPA accumulated in TUHR14TKB cells that expressed FABP7 (Fig. 5a and Additional file 3: Table S1). Ligand-binding studies conducted in vitro show that ω-3 EPA is the preferred ligand of FABP7 [33]. Further, the addition of docosatetraenoic acid significantly increased cell growth (Fig. 5b), suggesting that inhibition of the proliferation by FABP7 of TUHR14TKB cells does not act through the accumulation of docosatetraenoic acid by FABP7.

## Conclusions

Our data lead us to conclude that the TUHR14TKB cell line comprises a heterogeneous population and that cells that do not express FABP7 grow faster and are therefore selected during passage in culture. Further, our finding that FABP7 inhibited the proliferation of TUHR14TKB cells but stimulated the proliferation of 786-O cells cultured in medium with 1% FBS indicates that FABP7 function depends on cell type and culture conditions.

## Additional files

Additional file 1: Figure S1. Effect of FABP7 overexpression on the 786-O cell line. 786-O cells were cultured for two days in RPMI 1640 medium containing 10% FBS, 5 mg/L blasticidin S HCl, 0.3 g/L G418, and 1 mg/L doxycycline hyclate. **a**, Western blot analysis of FABP7 expression by cells transfected with the FABP7 vector or control (lacZ) vector. **b**, Real-time PCR analysis of FABP7 expression of cells transfected with the FABP7 vector or lacZ vector. **c**-**d**, The 786-O transfectants were cultured in RPMI-1640 medium containing 10% FBS or 1% FBS with 5 mg/L blasticidin S HCl, 0.3 g/L G418, and 1 mg/L doxycycline hyclate and subjected to cell proliferation and migration assays. **c**, The doubling times of cell transfected with the FABP7- or the lacZ-expression vector were determined using an MTS assay. The data represent the average and standard deviation (error bars) of five experiments. **d**, The migration of 786-O cells transfected with the FABP7- or lacZ-expression vector was determined using a wound-healing assay. The data represent the average and standard deviation (error bars) of four experiments. (TIFF 2702 kb)
Additional file 2: Figure S2. Proliferation of TUHR14TKB cells transfected with an FABP7 expression vector. The proliferation of cells transfected with the FABP7 expression vector or lacZ expression vector was determined using an MTS assay. The data represent of five experiments. Transfectants were cultured in RPMI 1640 medium containing 5 mg/L blasticidin S HCl, 0.3 g/L G418, and 1 mg/L doxycycline hyclate with 1% FBS (**a**-**b**) or 10% FBS (**c**-**d**). **a**, **c**, TUHR14TKB lacZ. **b**, **d**, TUHR14TKB FABP7. (TIFF 1521 kb)
Additional file 3: Table S1. Fatty acid concentrations of TUHR14TKB transfectants. The concentrations of fatty acids accumulated by TUHR14TKB cells transfected with vectors expressing FABP7 or lacZ were assayed in four independent cultures. (XLSX 46 kb)

## Abbreviations

cDNA: Complementary DNA
EPA: Eicosapentaenoic acid
FABP7: Fatty acid binding protein 7
FBS: Fetal bovine serum
lacZ: β-galactosidase
MTS: 3-(4,5-dimethylthiazol-2-yl)-5-(3-carboxymethoxyphenyl)-2-(4-sulfophenyl)-2H–tetrazolium
PBS: Phosphate-buffered saline
RCC: Renal cell carcinoma
SDS: Sodium dodecyl sulfate
siRNA: Small interfering RNA
TBP: TATA box binding protein

## References

[1] Ferlay J, Shin HR, Bray F, Forman D, Mathers C, Parkin DM (2010). Estimates of worldwide burden of cancer in 2008: GLOBOCAN 2008. Int J Cancer.

[2] Chow WH, Dong LM, Devesa SS (2010). Epidemiology and risk factors for kidney cancer. Nat Rev Urol.

[3] Pastore AL, Palleschi G, Silvestri L, Moschese D, Ricci S, Petrozza V (2015). Serum and urine biomarkers for human renal cell carcinoma. Dis Markers.

[4] Domoto T, Miyama Y, Suzuki H, Teratani T, Arai K, Sugiyama T (2007). Evaluation of S100A10, annexin II and B-FABP expression as markers for renal cell carcinoma. Cancer Sci.

[5] Shimizu F, Watanabe TK, Shinomiya H, Nakamura Y, Fujiwara T (1997). Isolation and expression of a cDNA for human brain fatty acid-binding protein (B-FABP). Biochim Biophys Acta.

[6] Seliger B, Lichtenfels R, Atkins D, Bukur J, Halder T, Kersten M (2005). Identification of fatty acid binding proteins as markers associated with the initiation and/or progression of renal cell carcinoma. Proteomics.

[7] Skubitz KM, Zimmermann W, Kammerer R, Pambuccian S, Skubitz AP (2006). Differential gene expression identifies subgroups of renal cell carcinoma. J Lab Clin Med.

[8] Perroud B, Lee J, Valkova N, Dhirapong A, Lin PY, Fiehn O (2006). Pathway analysis of kidney cancer using proteomics and metabolic profiling. Mol Cancer.

[9] Teratani T, Domoto T, Kuriki K, Kageyama T, Takayama T, Ishikawa A (2007). Detection of transcript for brain-type fatty Acid-binding protein in tumor and urine of patients with renal cell carcinoma. Urology.

[10] Tölle A, Jung M, Lein M, Johannsen M, Miller K, Moch H (2009). Brain-type and liver-type fatty acid-binding proteins: new tumor markers for renal cancer?. BMC Cancer.

[11] Raimondo F, Salemi C, Chinello C, Fumagalli D, Morosi L, Rocco F (2012). Proteomic analysis in clear cell renal cell carcinoma: identification of differentially expressed protein by 2-D DIGE. Mol BioSyst.

[12] Feng JY, Diao XW, Fan MQ, Wang PX, Xiao Y, Zhong X (2013). Screening of feature genes of the renal cell carcinoma with DNA microarray. Eur Rev Med Pharmacol Sci.

[13] Tan C, Takayama T, Takaoka N, Fujita H, Miyazaki M, Sugiyama T, Ozono S (2014). Impact of Gender in Renal Cell Carcinoma: The Relationship of FABP7 and BRN2 Expression with Overall Survival. Clin Med Insights Oncol.

[14] Zhou J, Deng Z, Chen Y, Gao Y, Wu D, Zhu G (2015). Overexpression of FABP7 promotes cell growth and predicts poor prognosis of clear cell renal cell carcinoma. Urol Oncol.

[15] Shi YE, Ni J, Xiao G, Liu YE, Fuchs A, Yu G (1997). Antitumor activity of the novel human breast cancer growth inhibitor, mammary-derived growth inhibitor-related gene. MRG Cancer Res.

[16] Wang M, Liu YE, Ni J, Aygun B, Goldberg ID, Shi YE (2000). Induction of Mammary Differentiation by Mammary-derived Growth Inhibitor- related Gene That Interacts with an omega-3 Fatty Acid on Growth Inhibition of Breast Cancer Cells. Cancer Res.

[17] Goto Y, Matsuzaki Y, Kurihara S, Shimizu A, Okada T, Yamamoto K (2006). A new melanoma antigen fatty acid-binding protein 7, involved in proliferation and invasion, is a potential target for immunotherapy and molecular target therapy. Cancer Res.

[18] Slipicevic A, Jørgensen K, Skrede M, Rosnes AK, Trøen G, Davidson B, Flørenes VA (2008). The fatty acid binding protein 7 (FABP7) is involved in proliferation and invasion of melanoma cells. BMC Cancer.

[19] Tölle A, Krause H, Miller K, Jung K, Stephan C (2011). Importance of brain-type fatty acid binding protein for cell-biological processes in human renal carcinoma cells. Oncol Rep.

[20] Liu RZ, Graham K, Glubrecht DD, Lai R, Mackey JR, Godbout R (2012). A fatty acid-binding protein 7/RXRβ pathway enhances survival and proliferation in triple-negative breast cancer. J Pathol.

[21] De Rosa A, Pellegatta S, Rossi M, Tunici P, Magnoni L, Speranza MC (2012). A radial glia gene marker, fatty acid binding protein 7 (FABP7), is involved in proliferation and invasion of glioblastoma cells. PLoS One.

[22] Mita R, Coles JE, Glubrecht DD, Sung R, Sun X, Godbout R (2007). B-FABP-expressing radial glial cells: the malignant glioma cell of origin?. Neoplasia.

[23] Mita R, Beaulieu MJ, Field C, Godbout R (2010). Brain fatty acid-binding protein and omega-3/omega-6 fatty acids: mechanistic insight into malignant glioma cell migration. J Biol Chem.

[24] Liang Y, Diehn M, Watson N, Bollen AW, Aldape KD, Nicholas MK (2005). Gene expression profiling reveals molecularly and clinically distinct subtypes of glioblastoma multiforme. Proc Natl Acad Sci U S A.

[25] Liang Y, Bollen AW, Aldape KD, Gupta N (2006). Nuclear FABP7 immunoreactivity is preferentially expressed in infiltrative glioma and is associated with poor prognosis in EGFR-overexpressing glioblastoma. BMC Cancer.

[26] RIKEN BioResource Center: Pattern of STR-PCR of TUHR14TKB. http://www2.brc.riken.jp/lab/cell/str_start.shtml?cell_no=RCB1383. Accessed 15 July 2016.

[27] Takaoka N, Takayama T, Teratani T, Sugiyama T, Mugiya S, Ozono S (2011). Analysis of the regulation of fatty acid binding protein 7 expression in human renal carcinoma cell lines. BMC Mol Biol.

[28] Jung M, Ramankulov A, Roigas J, Johannsen M, Ringsdorf M, Kristiansen G, Jung K (2007). In search of suitable reference genes for gene expression studies of human renal cell carcinoma by real-time PCR. BMC Mol Biol.

[29] de Wit NJ, Rijntjes J, Diepstra JH, van Kuppevelt TH, Weidle UH, Ruiter DJ, van Muijen GN (2005). Analysis of differential gene expression in human melanocytic tumour lesions by custom made oligonucleotide arrays. Br J Cancer.

[30] Goto Y, Koyanagi K, Narita N, Kawakami Y, Takata M, Uchiyama A (2010). Aberrant fatty acid-binding protein-7 gene expression in cutaneous malignant melanoma. J Invest Dermatol.

[31] Kinzler KW, Vogelstein B (1996). Lessons from hereditary colorectal cancer. Cell.

[32] Chaffer CL, Weinberg RA (2015). How Does Multistep Tumorigenesis Really Proceed?. Cancer Discov.

[33] Balendiran GK, Schnutgen F, Scapin G, Borchers T, Xhong N, Lim K (2000). Crystal structure and thermodynamic analysis of human brain fatty acid-binding protein. J Biol Chem.

[34] DMC CorpDMC Corp. http://dmed.co.jp. Accessed 15 July 2016

