# Supplementary figures and images for: Functional analysis of fatty acid binding protein 7 and its effect on fatty acid of renal cell carcinoma cell lines
Source: BMC Cancer. 2017 Mar 14;17:192. doi: 10.1186/s12885-017-3184-x (PMC5351052; doi:10.1186/s12885-017-3184-x)

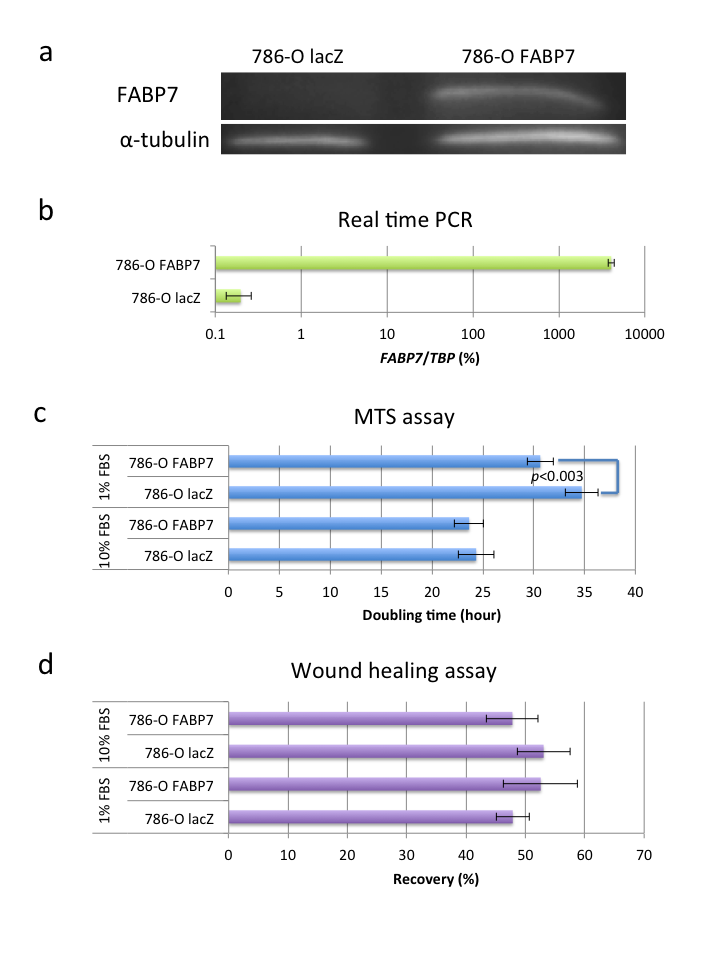

Supplement: Additional file 1: Figure S1. — Effect of FABP7 overexpression on the 786-O cell line. 786-O cells were cultured for two days in RPMI 1640 medium containing 10% FBS, 5 mg/L blasticidin S HCl, 0.3 g/L G418, and 1 mg/L doxycycline hyclate. a, Western blot analysis of FABP7 expression by cells transfected with the FABP7 vector or control (lacZ) vector. b, Real-time PCR analysis of FABP7 expression of cells transfected with the FABP7 vector or lacZ vector. c-d, The 786-O transfectants were cultured in RPMI-1640 medium containing 10% FBS or 1% FBS with 5 mg/L blasticidin S HCl, 0.3 g/L G418, and 1 mg/L doxycycline hyclate and subjected to cell proliferation and migration assays. c, The doubling times of cell transfected with the FABP7- or the lacZ-expression vector were determined using an MTS assay. The data represent the average and standard deviation (error bars) of five experiments. d, The migration of 786-O cells transfected with the FABP7- or lacZ-expression vector was determined using a wound-healing assay. The data represent the average and standard deviation (error bars) of four experiments. (TIFF 2702 kb) [file 12885_2017_3184_MOESM1_ESM.tiff]

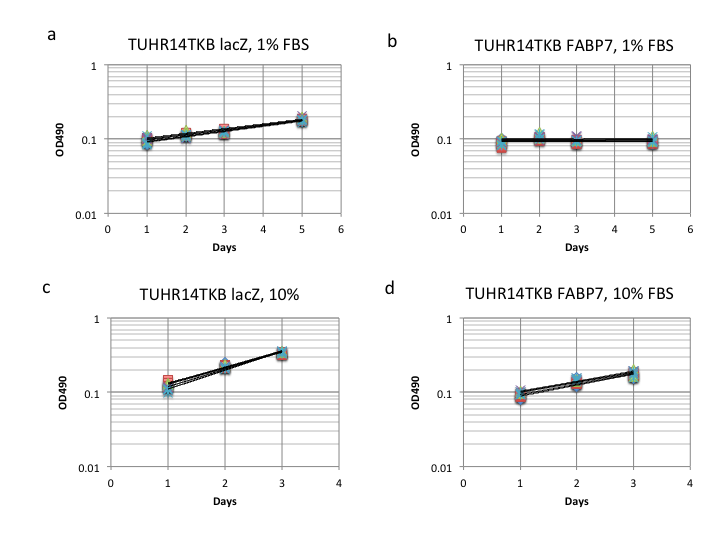

Supplement: Additional file 2: Figure S2. — Proliferation of TUHR14TKB cells transfected with an FABP7 expression vector. The proliferation of cells transfected with the FABP7 expression vector or lacZ expression vector was determined using an MTS assay. The data represent of five experiments. Transfectants were cultured in RPMI 1640 medium containing 5 mg/L blasticidin S HCl, 0.3 g/L G418, and 1 mg/L doxycycline hyclate with 1% FBS (a-b) or 10% FBS (c-d). a, c, TUHR14TKB lacZ. b, d, TUHR14TKB FABP7. (TIFF 1521 kb) [file 12885_2017_3184_MOESM2_ESM.tiff]
